# Relationship between tumor mutational burden and maximum standardized uptake value in 2-[^18^F]FDG PET (positron emission tomography) scan in cancer patients

**DOI:** 10.1186/s13550-020-00732-z

**Published:** 2020-12-09

**Authors:** Amin Haghighat Jahromi, Donald A. Barkauskas, Matthew Zabel, Aaron M. Goodman, Garret Frampton, Mina Nikanjam, Carl K. Hoh, Razelle Kurzrock

**Affiliations:** 1 Department of Radiology, University of California, San Diego, La Jolla, CA USA; 2 Department of Preventive Medicine, Biostatistics Division, Keck School of Medicine of the University of Southern California, Los Angeles, CA USA; 3 Division of Blood and Marrow Transplantation, Department of Medicine, University of California San Diego (UCSD), La Jolla, CA USA; 4 Foundation Medicine, Cambridge, MA USA; 5 Center for Personalized Cancer Therapy and Division of Hematology and Oncology, Department of Medicine, University of California San Diego Moores Cancer Center, La Jolla, CA USA

**Keywords:** Tumor mutational burden, SUV_max_, Cancer, Immunotherapy

## Abstract

**Purpose:**

Deriving links between imaging and genomic markers is an evolving field. 2-[^18^F]FDG PET/CT (^18^F-fluorodeoxyglucose positron emission tomography–computed tomography) is commonly used for cancer imaging, with maximum standardized uptake value (SUV_max_) as the main quantitative parameter. Tumor mutational burden (TMB), the quantitative variable obtained using next-generation sequencing on a tissue biopsy sample, is a putative immunotherapy response predictor. We report the relationship between TMB and SUV_max_, linking these two important parameters.

**Methods:**

In this pilot study, we analyzed 1923 patients with diverse cancers and available TMB values. Overall, 273 patients met our eligibility criteria in that they had no systemic treatment prior to imaging/biopsy, and also had 2-[^18^F]FDG PET/CT within 6 months prior to the tissue biopsy, to ensure acceptable temporal correlation between imaging and genomic evaluation.

**Results:**

We found a linear correlation between TMB and SUV_max_ (*p* < 0.001). In the multivariate analysis, only TMB independently correlated with SUV_max_, whereas age, gender, and tumor organ did not.

**Conclusion:**

Our observations link SUV_max_ in readily available, routinely used, and noninvasive 2-[^18^F]FDG PET/CT imaging to the TMB, which requires a tissue biopsy and time to process. Since higher TMB has been implicated as a prognostic biomarker for better outcomes after immunotherapy, further investigation will be needed to determine if SUV_max_ can stratify patient response to immunotherapy.

## Introduction

Creating a link between imaging findings and genomic data in patients with cancer is crucial in the evolving world of genomics. Radiologic markers have shown promise for noninvasive identification of molecular properties [1]. Imaging markers can provide surrogate genomic markers from imaging data for diagnosis, prognosis, and stratification of cancer patients in the emerging field of personalized medicine. Such links between imaging and genomics have been formed in computed tomography (CT) and magnetic resonance imaging (MRI), whereas fewer studies have investigated such relationship in 2-[^18^F]fluoro-2-deoxy-d-glucose (2-[^18^F]FDG) positron emission tomography (PET) [2–4].

2-[^18^F]FDG PET/CT is standard of care and plays a pivotal role in cancer diagnosis and staging [5]. The maximum standardized uptake value (SUV_max_), a relative measure of FDG uptake, is the most widely used quantitative parameter for the assessment of cancer patients [6, 7]. Traditionally, the SUV_max_ has been correlated with histopathological findings. As the era of personalized medicine continues to move rapidly toward molecular stratification, there have been studies to associate SUV_max_ with biologic pathways, although SUV_max_ is still generally unspecified at a molecular level [4, 8].

Tumor mutational burden (TMB) is defined as the total number of somatic mutations identified per megabase pair (Mbp) of coding area and is quantified using next-generation sequencing (NGS). Tumors with high TMB likely harbor numerous neoantigens created from the mutanome, possibly eliciting an endogenous immune response or enhancing tumor metabolic rate, which may explain the correlation of high TMB and response to checkpoint blockade immunotherapy [9–12].

Herein, we demonstrate a positive relationship between SUV_max_ and TMB in patients with cancer. Our finding links SUV_max_ in readily available, routinely used, and noninvasive 2-[^18^F]FDG PET/CT imaging to the TMB, which requires a tissue biopsy and time to process. Since higher TMB has been suggested to predict better outcomes after immunotherapy, further prospective study of this association and its implications for cancer immunotherapy are needed.

## Results

### Patient characteristics

Of 1923 patients in the database, we found 273 patients with metastatic cancer had no systemic treatment prior to imaging/biopsy, and also had 2-[^18^F]FDG PET/CT within 6 months prior to the tissue biopsy (Table 1). All patients had metastatic (stage IV) disease at the time of blood draw. Because patients with early-stage cancer were not included in the study, the cancer stage was not a variable. Based on the TMB values and prior precedent for cut-off values [13], patients were categorized into three groups: TMB = 0–1 mutations/mb (*N* = 39 patients, 14%), TMB = 2–11 mutations/mbp (*N* = 174, 64%), and TMB ≥ 12 mutations/mb (*N* = 60, 22%); the mean TMB in each group was 0, 5, and 37 mutations/mbp. There was no difference in gender distribution between groups. Patients in the TMB = 2–11 mutations/mb group were slightly younger on average than those in the two other TMB groups (63.9 vs. 68.5 or 68.3 years, *p* = 0.03). Organ distribution showed statistically significant differences between TMB categories in different cancer types, with higher TMB in melanoma and lung cancer than in breast, gastrointestinal, or “other” cancers (Table 1). The most notable pattern was seen with melanoma, which expectedly had the highest percentage of patients among cancer types in the TMB ≥ 12 mutations/mb category (60% vs. 7–30% in other cancer types) [12, 14].

**Table 1:** Patient characteristics in three quantiled TMB groups (N = 273 patients)

	TMB (0–1 mutations/mb) *N* = 39	TMB (2–11 mutations/mb) *N* = 174	TMB (≥ 12 mutations/mb) *N* = 60	*p* value
TMB (Mean ± SD)	0	5.2 ± 2.5	37.4 ± 47.6	Not applicable
Median	0	5	20	
*SUV*_*max*_* (Mean ± SD)*	*4.5 ± 3.9*	*8.4 ± 7.8*	*11.2 ± 8.5*	*p < 0.0001*
Median (range)	3.9 (0–16.4)	6.8 (0–74.0)	9.2 (1.6–49.6)	
Age at time of biopsy (years) (Mean ± SD)	68.5 ± 12.5	63.9 ± 13.7	68.3 ± 12.8	*p = 0.03*
Median (range)	71 (34–91)	65 (23–96)	70 (28–89)	
Women (*N* (%))	22 (14%)	104 (67%)	30 (19%)	p = 0.4
Men (*N* (%))	17 (14%)	70 (60%)	30 (26%)	
Melanoma (*N* = 15)	2 (13%)	4 (27%)	9 (60%)	*p < 0.001*
Lung cancer (*N* = 61)	5 (8%)	38 (62%)	18 (30%)	
Gastrointestinal (*N* = 36)	3 (9%)	29 (80%)	4 (11%)	
Breast (*N* = 43)	7 (16%)	33 (77%)	3 (7%)	
Other (*N* = 118)*	22 (19%)	70 (59%)	26 (22%)	

*SD* standard deviation, *SUV* standardized uptake value, *TMB* tumor mutational burden

^*^Other cancers consisted of head and neck, adrenal, bladder, ovary, uterus, prostate, musculoskeletal, and hematologic malignancies, and cancers of unknown primary

### ***SUV***_***max***_*** correlates with TMB***

**Fig. 1:**
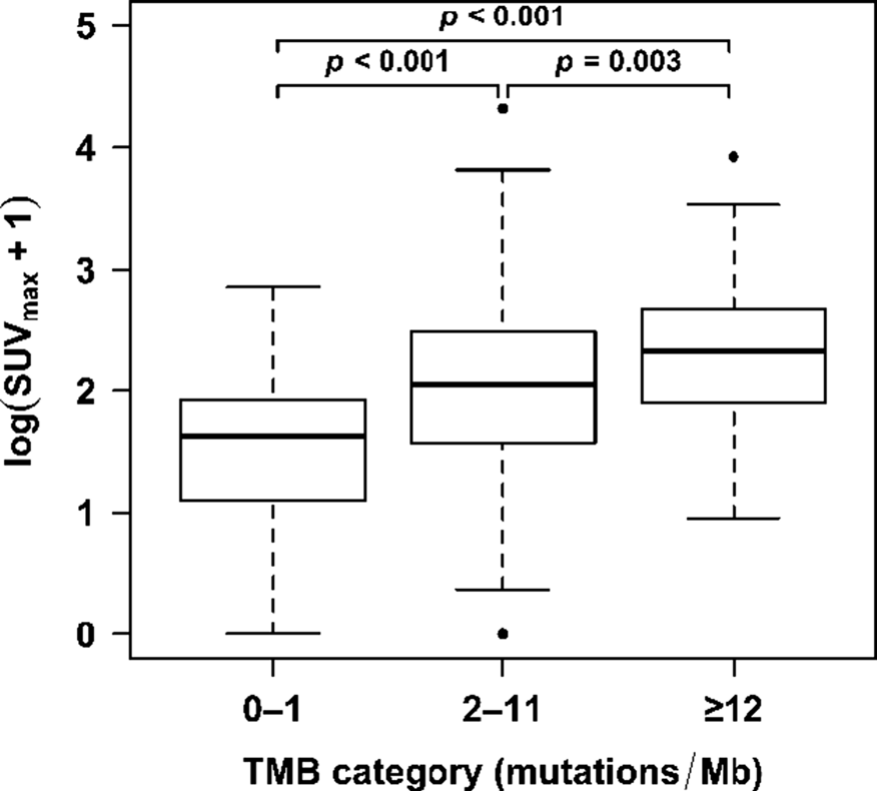
SUV_max_ is significantly different between TMB of 0–1, 2–11, and ≥ 12 mutations/mb. The central thick black line indicates the median, and the bottom and top of the rectangle are the 25th (Q1) and 75th (Q3) percentiles. The circles represent outlier SUV_max_ values, defined as either larger than Q3 + 1.5 × IQR or smaller than Q1 − 1.5 × IQR, where IQR = Q3 − Q1 is the interquartile range. The horizontal “whiskers” represent the largest and smallest non-outlier observations in the data set. All *p* values are from analysis on log scale

**Fig. 2:**
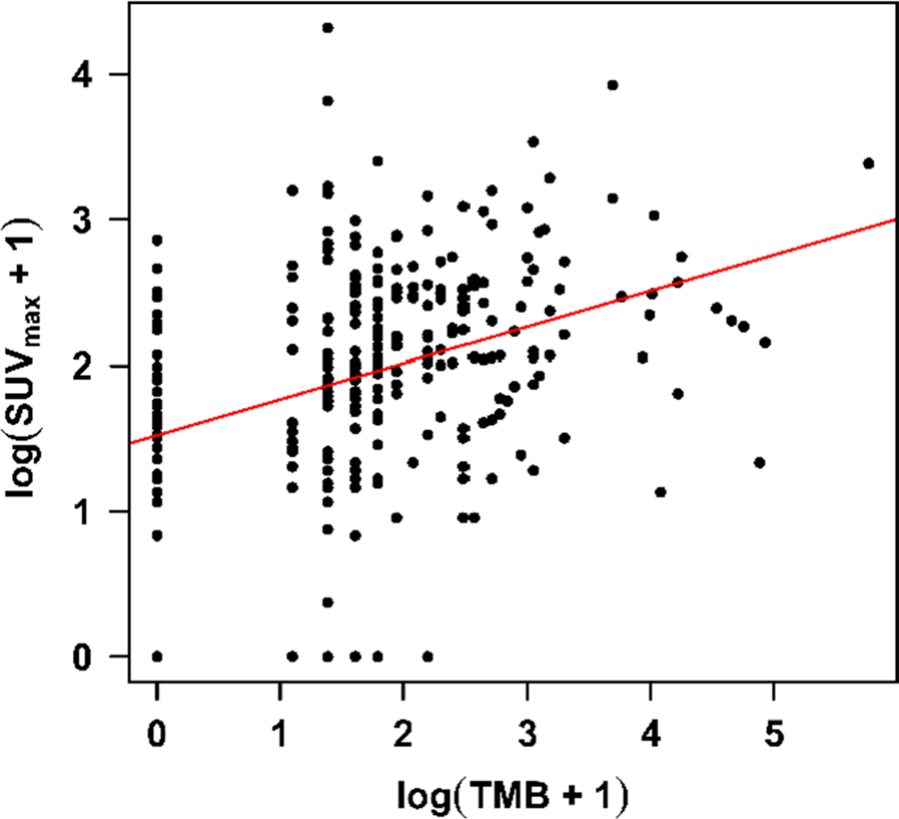
log(SUV_max_ + 1) is linearly correlated to log(TMB + 1) (*r* = 0.34, *p* < 0.001). The graph is the regression on the shifted-log scale. The circles represent individual data-points

Median SUV_max_ was 3.9, 6.8 and 9.2 for the 0–1, 2–11, and ≥ 12 mutations/mb groups (*p* < 0.0001). Raw diagnostics showed that the TMB is the only variable with statistically significant relationship with SUV_max_ (*p* < 0.003) (Table 2). However, due to the highly skewed distributions of both TMB and SUV, shifted-log transformations were used for analysis because statistical model diagnostics indicated that both SUV_max_ and TMB should be analyzed on the log scale (Table 3). Post hoc analysis showed significant differences between SUV_max_ in all groups: mean increase in shifted SUV_max_ values for TMB ≥ 12 mutations/mb category vs. TMB 2–11 category was 38.6% with 95% CI = [11.7%,71.9%] (*p* < 0.003); mean increase in shifted SUV_max_ values for TMB ≥ 12 category vs. TMB 0–1 category was 145% with 95% CI = [82.2%, 229.5%] (*p* < 0.001), and for TMB 2–11 category vs. TMB 0–1 category was 76.8%, with 95% CI = [37.0%,128.2%], (*p* < 0.001) (Fig. 1). Linear correlation between all shifted-log TMB and shifted-log SUV_max_ had Pearson correlation coefficient *r* = 0.34, (*p* < 0.001) (Fig. 2). Among different cancer types, breast cancer patients showed linear correlation between shifted-log TMB and shifted-log SUV_max_ with Pearson correlation coefficient *r* = 0.40, (*p* = 0.008). This linear correlation coefficient for the lung cancer patients was *r* = 0.43, (*p* = 0.001), and for the other cancer patients was *r* = 0.37 (*p* < 0.001). For the melanoma and gastrointestinal patients, this relationship was not statistically significant, perhaps due to smaller number of patients in these two groups (*N* = 15, and 36, respectively).

**Table 2:** Univariate analysis of relationship of variables to SUV_max_ in the raw scale

	Unit increase in SUV_max_ compared to reference, univariate model (95% CI)	*P* value univariate
TMB	0.06 (0.02, 0.09)	*0.002*
Age (years)	0.04 (− 0.03, 0.11)	*0.3*
Men (*N* = 117)	− 0.40 (− 2.27, 1.47)	*0.7*
Women (*N* = 156)	Reference	
Melanoma (*N* = 15)	1.97 (− 2.73, 6.67)	*0.4*
Lung cancer (*N* = 61)	2.20 (− 1.02, 5.41)	
Gastrointestinal (*N* = 36)	Reference	
Breast (*N* = 43)	− 0.58 (− 4.03, 2.88)	
Other (*N* = 118)	0.77 (− 2.14, 3.68)	

^*^Higher TMB was significantly correlated with increased SUV_max_

**Table 3:** Multivariate analysis of relationship of variables to SUV_max_ in the log scale*

	Percent increase in (SUV_max_ + 1) per unit increase in variable, univariate model (95% CI)*	*p* value univariate	Percent increase in (SUV_max_ + 1) per unit increase in variable, multivariate model (95% CI)*	*p* value multivariate***
Log(TMB + 1)	27.9% (18.0%, 38.7%)**	*< 0.001*	27.8% (17.8%, 38.7%)	*< 0.001*
Age (years)	0.2% (− 0.4%, 0.9%)**	0.5	–	–
Men (*N* = 117)	4.3% (− 13.5%, 25.7%)	0.7	–	–
Women (*N* = 156)	Reference			
Melanoma (*N* = 15)	12.6% (− 29.3%, 79.2%)	0.07	–3.5% (–37.9%, 50.0%)	*0.08*
Lung cancer (*N* = 61)	48.8% (8.3%, 104.5%)		45.1% (7.5%, 95.8%)	
Gastrointestinal (*N* = 36)	Reference		Reference	
Breast (*N* = 43)	3.3% (− 26.6%, 45.5%)		13.3% (− 18.0%, 56.6%)	
Other (*N* = 118)	13.6% (− 14.8%, 51.6%)		17.1% (− 10.7%, 53.7%)	

^*^Statistical model diagnostics indicated that SUV_max_ and TMB should be analyzed on the log scale, due to the highly skewed distributions of both TMB and SUV_max_. TMB and SUV_max_ values are analyzed as linear variables on shifted-log scale. *CI* confidence interval

^**^For every 1 unit increase in log(TMB + 1), there is a 27.9% increase in the predicted geometric mean. Similarly, for every year increase in age, there is a 0.2% increase in the predicted geometric mean. See “Methods” section for statistical analysis

^***^Only variables with *p* value ≤ 0.1 in univariate were tested in multivariate analysis

^****^Higher log(TMB + 1) was significantly correlated with increased (SUV_max_ + 1)

### ***Multivariate analysis of factors affecting SUV***_***max***_

We found that sex and age had no correlation with SUV_max_. Cancer type relationship with SUV_max_ showed a statistically insignificant trend (with lung cancer having higher SUV_max_, albeit not statistically significant) (multivariate *p* value = 0.08). The only variable that correlated significantly with SUV_max_, was TMB; Each unit increase in log(TMB + 1) resulted in a 27.8% increase in (SUV_max_ + 1) (multivariate *p* < 0.001) (Table 3).

## Discussion

To our knowledge, this is the first study to investigate the relationship between SUV_max_ and TMB in patients with diverse cancers. Prior studies have investigated the complex relationship between tumor immune microenvironment and glucose metabolism rate and shown associations between metabolic and immune profiles [15, 16]. 2-[^18^F]FDG PET imaging has been suggested as a diagnostic tool to estimate tumor immune status [17]. Our hypothesis was that higher mutational load (as reflected by TMB) might correlate with metabolic reconfiguration [18], and immune inflammatory response [12], and that either of these features would be associated with a higher SUV_max_ [19]. Our study confirmed that higher TMB was the only evaluated variable that independently correlated with higher SUV_max_. Of interest, one prior study examined this question, albeit in lung cancer alone [4]. They found no significant relationship between SUV_max_ and TMB. However, there were some major differences between their study and ours: (1) Moon and colleagues confined their observations to lung cancer, whereas our study included a variety of malignancies; and (2) they did not note the timing of the 2-[^18^F]FDG PET–CT versus the biopsy [4]. In our study, the biopsy was taken ≤ 6 months before the PET scan. Longer time lapse may cause poor correlation between the SUV_max_ and TMB parameters. In addition, obtaining PET imaging after the biopsy or after starting the treatment could cause false positive or false negative SUV_max_ results. Indeed, we studied 1923 diverse cancer patients with TMB values, out of which 273 patients met the criteria of having no prior systemic treatment and having SUV_max_ performed within 6 months prior to the biopsy. Since having synchronous TMB and SUV_max_ is ideal, future studies should attempt to obtain biopsies for TMB immediately after PET imaging.

Various cutoffs have been previously established for TMB, including the dichotomization at 12 mutations/mb, to be predictive of immunotherapy response [13]. Upon categorizing patients into three groups based on TMB levels: 0–1, 2–11, and ≥ 12 mutations/mb groups, we found that patients in the higher TMB group have higher SUV_max_ values and this difference was statistically significant between all three groups with *p* < 0.0001 (Table 1).

To confirm that the relationship between TMB and SUV_max_ is independent of confounders, we analyzed the data in multivariate models. The only parameter that showed a significant relationship with SUV_max_ was TMB (multivariate *p* < 0.0001), confirming the independent correlation between TMB and SUV_max_ (Table 3). Further, there was a linear relationship between TMB and SUV_max_ in the log scale (*r* = 0.34, *p* < 0.001) (Fig. 2). Sex, age, and cancer type had no statistically significant association with SUV_max_ (Table 3).

Several genomic alterations have been related to immunotherapy response, including but not limited to microsatellite instability high (MSI-H) status (which results in high TMB), high TMB itself, *PBRM1* mutations, and APOBEC-related mutagenesis [12, 13, 20–22]. TMB varies dramatically between tumor types, with skin and lung cancers, having higher median TMBs than most other cancers [23, 24]. Our previous studies indicated that the median TMB for responders vs. non-responders to anti-PD-1/PD-L1 monotherapy was 18.0 vs. 5.0 mutations/Mb, with higher TMB predicting favorable outcomes across diverse tumors [12, 25]. Other studies have found that higher TMB was linked to improved survival following immunotherapy in diverse cancers for the top 20% of TMBs in each histology [26]. Various investigations have used different cutoffs for defining the relationship between TMB and checkpoint blockade response [27] and our own work has suggested a linear correlation between TMB and response [12].

We hypothesize that higher TMB promotes metabolic reconfiguration, causing increased glucose metabolism rate (GMR), and thus higher SUV_max_. Carbohydrate metabolism has been previously shown to have correlation with TMB [18]. GMR–TMB correlation could explain our finding of SUV_max_–TMB correlation, although the exact mechanism for this finding is not understood [18]. An alternative explanation for the correlation between TMB and SUV_max_ might be based on an innate immune response to tumors with higher TMB. Indeed, higher TMB correlates with better response to immune checkpoint blockade and it is conceivable that innate immunity might also be triggered in the presence of high mutational load. An immune cell infiltrate would create increased glycolytic activity and an inflammatory response that would manifest as higher SUV_max_ [28]. We have also previously shown increased SUV_max_ in tumors with higher number of characterized genomic alterations [29, 30] consistent with this work.

Our study had several important limitations: first it is a retrospective analysis and thus TMB and SUV_max_ parameters were not fully synchronized; second, although the full cohort included 1923 patients, only 273 patients had PET scans within 6 months before their biopsies for TMB; third, we do not know the mechanism underlying the relationship between TMB and SUV_max;_ fourth this study was single-center/single-camera, and fifth, a variety of tumor types were included in the analysis, though the latter two may also suggest the homogeneity of PET results and generalizability of results across cancer organs, respectively); and fourth, we did not examine variant genes or molecules associated with SUV_max_, which could be key markers of SUV_max_ [31]*.* Future studies are needed to expand the number of the patients, and to evaluate such relationship in each individual cancer type. Multicenter study and also same-day PET scans/biopsy for TMB are needed would be needed to validate our findings. Furthermore, exploring the direct relationship between SUV_max_ and immunotherapy response is future step since we found SUV_max_ is correlated with TMB and it is known that higher TMB is correlated with immunotherapy response.

## Materials and methods

### Patient selection

We performed a search and found 1923 patients who had TMB values on biopsy tissue samples obtained by hybrid capture-based NGS (Foundation Medicine) at UC San Diego Moores Cancer Center. Among those, 273 patients had no systemic treatment prior to imaging/biopsy, and also had 2-[^18^F]FDG PET/CT within 6 months prior to the tissue biopsy, to ensure acceptable temporal correlation between imaging and genomic evaluation.

### 2***-***[^18^F]FDG PET–CT imaging

All patients received PET imaging under standard conditions as needed for their disease assessment. Patients were asked to fast for at least 6 h prior to their scan. Blood glucose levels were measured immediately before the 2-[^18^F]FDG injection and no patient had a blood glucose level > 8.9 mM. Patients were injected with 370–740 MBq 2-[^18^F]FDG, intravenously, within 5–10 s. Following an uptake period of approximately one hour in a quiet room at rest, multi-station 3-dimensional (3D) whole-body PET acquisition with CT (for attenuation correction) was performed for approximately 60 min, using the same GE Discovery VCT scanner (GE, Waukesha, WI) for all the patients. The scanner was in compliance with American College of Radiology guidelines. Whole-body CT covered the region from the head to the mid-thigh. PET images were acquired, after the CT scan, at a rate of 2 min/bed position, in the 3 dimensional (3D) acquisition mode. CT images were then reconstructed onto a 512 × 512 matrix. PET images were reconstructed using a standard whole-body 3D iterative reconstruction: 2 iterations; 28 subsets onto a 128 × 128 matrix with attenuation correction, decay correction, and scatter correction. The photon energy window was 425–650 keV. Slice thickness was 3.27 mm and reconstruction diameter was 70 cm. Pixel size was 5.47 mm × 5.47 mm with spatial resolution of 5 mm. 2-[^18^F]FDG PET/ CT images were generated for review on a workstation.

### Image analysis

All PET images were interpreted on the institution’s pictures archiving and communication system (PACS), (AGFA Impax 6.3, Mortsel Belgium) by a board-certified academic nuclear medicine physician/radiologist and verified by a second nuclear medicine physician/radiologist. Focal activities of the lesions were manually identified on the PET images. SUVs of the lesions were obtained by manually placing a circular region of interest (ROI) at the site of the maximum 2-[^18^F]FDG uptake in the PET images and the maximal activity (SUV_max_) was recorded. The SUV was calculated as decay-corrected activity of tissue volume (kBq /mL)/injected 2-[^18^F]FDG activity per body mass (kBq/g). In most of the cases, the biopsied lesion was selected for analysis; however, if the biopsied lesion was smaller than 1 cm, the most FDG-avid lesion, larger than 1 cm, was selected, to avoid partial volume effect. Therefore, all the lesions that underwent SUV_max_ analysis, were > 1 cm diameter. For patients showing no focal 2-[^18^F]FDG uptake on PET, a rounded SUV_max_ of 0 was recorded. It should be noted that those patients with only background uptake have no elevated glucose uptake; the exact SUV_max_ number may vary in these different patients due to technique and background, so they were all rounded to 0, for a more accurate representation.

### Evaluation of TMB

Formalin-fixed, paraffin-embedded tumors were submitted for NGS to Foundation Medicine (clinical laboratory improvement amendments (CLIA)-certified lab). The Foundation One assay was used (hybrid-capture-based NGS; 182, 236, or 315 genes, depending on the time period). The methods have been previously described [32]. Average sequencing depth of coverage was > 250 × , with > 100 × at > 99% of exons. For TMB, the number of somatic mutations detected by interrogating 1.2 mb of the genome were quantified and that value extrapolated to the whole exome using a validated algorithm. Alterations likely or known to be oncogenic drivers as well as germline polymorphisms were excluded. TMB was measured in mutations per megabase pair (Mbp). TMB levels were divided into three groups based off the Foundation Medicine official reports: 0–1, 2–11, and ≥ 12 mutations/mb.

### Statistical analysis

Statistical analysis was done in R, version 4.0.2. Statistical model diagnostics indicated that both SUV_max_ and TMB should be analyzed on the log scale. Since there were multiple rounded zero values in both SUV_max_ and TMB values, they were transformed with a shifted-log by adding 1 before taking the natural logarithm. The TMB data were also grouped into three TMB quantiles (0–1, 2–11, and ≥ 12 mutations/Mb) for analysis as a categorical variable. The categorized TMB data were analyzed for association with the shifted-log SUV_max_ values by ANOVA, with sex by Fisher’s Exact test, and with cancer type by a chi-squared test. The shifted-log SUV_max_ was regressed on the shifted-log TMB and on age in years and was also used as the response variable in ANOVAs with sex and cancer type. The variables with *p* < 0.1 in these four analyses were then used in a general linear model with SUV_max_ as the response variable. Differences between groups were considered to be significant at a *p* value ≤ 0.05 and confidence intervals (CI) were done at confidence level 95%. The geometric mean was used for some analysis (geometric mean of N numbers is the nth root of the product of the numbers). Data are reported as mean ± standard deviation (SD).

## Conclusion

We found a linear positive correlation between TMB and SUV_max_ in diverse cancers. Of the features evaluated, multivariate analysis showed TMB to be the only factor independently associated with SUV_max_. Future prospective studies with PET scans and biopsy for TMB done on the same day are needed to validate the findings in this area. Furthermore, it will be important to determine if tumors with higher SUV_max_ respond better to immunotherapy, as might be expected, since higher TMB is correlated with immunotherapy response [12].

## Data Availability

Not applicable.

## Abbreviations

2-[^18^F]FDG PET/CT: 2-[^18^F]fluoro-2-deoxy-d-glucose positron emission tomography–computed tomography
SUV_max_: Standardized uptake value
TMB: Tumor mutational burden
MRI: Magnetic resonance imaging
Mbp: Megabase pair
NGS: Next-generation sequencing
PACS: Pictures archiving and communication system
ROI: Region of interest
GMR: Glucose metabolism rate

## References

[1] Jansen RW, van Amstel P, Martens RM, Kooi IE, Wesseling P, de Langen AJ (2018). Non-invasive tumor genotyping using radiogenomic biomarkers, a systematic review and oncology-wide pathway analysis. Oncotarget.

[2] Segal E, Sirlin CB, Ooi C, Adler AS, Gollub J, Chen X (2007). Decoding global gene expression programs in liver cancer by noninvasive imaging. Nat Biotechnol.

[3] Diehn M, Nardini C, Wang DS, McGovern S, Jayaraman M, Liang Y (2008). Identification of noninvasive imaging surrogates for brain tumor gene-expression modules. Proc Natl Acad Sci U S A.

[4] Moon SH, Kim J, Joung JG, Cha H, Park WY, Ahn JS (2019). Correlations between metabolic texture features, genetic heterogeneity, and mutation burden in patients with lung cancer. Eur J Nucl Med Mol Imaging.

[5] Fletcher JW, Djulbegovic B, Soares HP, Siegel BA, Lowe VJ, Lyman GH (2008). Recommendations on the use of 18F-FDG PET in oncology. J Nucl Med.

[6] Lodge MA (2017). Repeatability of SUV in oncologic. J Nucl Med.

[7] Thie JA (2004). Understanding the standardized uptake value, its methods, and implications for usage. J Nucl Med.

[8] Ahn KS, Kang KJ, Kim YH, Kim TS, Song BI, Kim HW (2019). Genetic features associated with ^18^F-FDG uptake in intrahepatic cholangiocarcinoma. Ann Surg Treat Res.

[9] Chan TA, Yarchoan M, Jaffee E, Swanton C, Quezada SA, Stenzinger A (2019). Development of tumor mutation burden as an immunotherapy biomarker: utility for the oncology clinic. Ann Oncol.

[10] Rizvi NA, Hellmann MD, Snyder A, Kvistborg P, Makarov V, Havel JJ (2015). Cancer immunology mutational landscape determines sensitivity to PD-1 blockade in non-small cell lung cancer. Science.

[11] Snyder A, Makarov V, Merghoub T, Yuan J, Zaretsky JM, Desrichard A (2014). Genetic basis for clinical response to CTLA-4 blockade in melanoma. N Engl J Med.

[12] Goodman AM, Kato S, Bazhenova L, Patel SP, Frampton GM, Miller V (2017). Tumor mutational burden as an independent predictor of response to immunotherapy in diverse cancers. Mol Cancer Ther.

[13] Goodman AM, Kato S, Chattopadhyay R, Okamura R, Saunders IM, Montesion M (2019). Phenotypic and genomic determinants of immunotherapy response associated with squamousness. Cancer Immunol Res.

[14] Maleki VS (2018). High and low mutational burden tumors versus immunologically hot and cold tumors and response to immune checkpoint inhibitors. J Immunother Cancer.

[15] Na KJ, Choi H (2018). Tumor metabolic features identified by ^18^F-FDG PET correlate with gene networks of immune cell microenvironment in head and neck cancer. J Nucl Med.

[16] Park C, Na KJ, Choi H, Ock CY, Ha S, Kim M (2020). Tumor immune profiles noninvasively estimated by FDG PET with deep learning correlate with immunotherapy response in lung adenocarcinoma. Theranostics.

[17] Togo M, Yokobori T, Shimizu K, Handa T, Kaira K, Sano T (2020). Diagnostic value of 18 F-FDG-PET to predict the tumour immune status defined by tumoural PD-L1 and CD8 + tumour-infiltrating lymphocytes in oral squamous cell carcinoma. Br J Cancer.

[18] Choi H, Na KJ (2018). Pan-cancer analysis of tumor metabolic landscape associated with genomic alterations. Mol Cancer.

[19] Zikou A, Sioka C, Alexiou GA, Fotopoulos A, Voulgaris S, Argyropoulou MI (2018). Radiation necrosis, pseudoprogression, pseudoresponse, and tumor recurrence: imaging challenges for the evaluation of treated gliomas. Contrast Media Mol Imaging.

[20] Mouw KW, Goldberg MS, Konstantinopoulos PA, D'Andrea AD (2017). DNA damage and repair biomarkers of immunotherapy response. Cancer Discov.

[21] Otto G (2018). Kidney cancer: PBRM1 loss promotes tumour response to immunotherapy. Nat Rev Clin Oncol.

[22] Boichard A, Pham TV, Yeerna H, Goodman A, Tamayo P, Lippman S (2019). APOBEC-related mutagenesis and neo-peptide hydrophobicity: implications for response to immunotherapy. Oncoimmunology.

[23] Chalmers ZR, Connelly CF, Fabrizio D, Gay L, Ali SM, Ennis R (2017). Analysis of 100,000 human cancer genomes reveals the landscape of tumor mutational burden. Genome Med.

[24] Alexandrov LB, Nik-Zainal S, Wedge DC, Aparicio SA, Behjati S, Biankin AV (2013). Signatures of mutational processes in human cancer. Nature.

[25] Goodman A, Patel SP, Kurzrock R (2017). PD-1-PD-L1 immune-checkpoint blockade in B-cell lymphomas. Nat Rev Clin Oncol.

[26] Samstein RM, Lee CH, Shoushtari AN, Hellmann MD, Shen R, Janjigian YY (2019). Tumor mutational load predicts survival after immunotherapy across multiple cancer types. Nat Genet.

[27] Hellmann MD, Ciuleanu TE, Pluzanski A, Lee JS, Otterson GA, Audigier-Valette C (2018). Nivolumab plus ipilimumab in lung cancer with a high tumor mutational burden. N Engl J Med.

[28] Aide N, Hicks RJ, Le Tourneau C, Lheureux S, Fanti S, Lopci E (2019). FDG PET/CT for assessing tumour response to immunotherapy : Report on the EANM symposium on immune modulation and recent review of the literature. Eur J Nucl Med Mol Imaging.

[29] Chang GH, Kurzrock R, Tran L, Schwaederle M, Hoh CK (2018). mutations and number of alterations correlate with maximum standardized uptake value (SUVmax) determined by positron emission tomography/computed tomography (PET/CT). Oncotarget.

[30] Haghighat Jahromi A, Chang C, Hoh CK, Kurzrock R (2020). Standardized uptake value (SUVmax) in ^18^F-FDG PET/CT is correlated with the total number of main oncogenic anomalies in cancer patients. Cancer Biol Ther..

[31] Kim SK, Ahn SG, Mun JY, Jeong MS, Bae SJ, Lee JS (2020). Genomic signature of the standardized uptake value in ^18^F-Fluorodeoxyglucose positron emission tomography in breast cancer. Cancers (Basel).

[32] Frampton GM, Fichtenholtz A, Otto GA, Wang K, Downing SR, He J (2013). Development and validation of a clinical cancer genomic profiling test based on massively parallel DNA sequencing. Nat Biotechnol.

